# Plantar pressure distribution during gait cycle after subtalar arthroereisis in adolescent flexible flatfoot

**DOI:** 10.3389/fped.2025.1618096

**Published:** 2025-11-18

**Authors:** Nu Xiong, Li Chen, TianHong Ru, Chao Zhang, JiaZhang Huang, Xu Wang, Xin Ma

**Affiliations:** 1 Huashan Hospital, Fudan University, Shanghai, China; 2 Shanghai Sixth People’s Hospital, Shanghai Jiao Tong University, Shanghai, China

**Keywords:** flexible flatfoot, subtalar arthroereisis, plantar pressure, surface electromyography, postoperative follow-up

## Abstract

**Background:**

Flexible flatfoot is a common foot deformity in adolescents. Subtalar arthroereisis can help reduce excessive foot pronation by placing an implant in the subtalar joint. In recent years, this method has been widely used to treat adolescent flexible flat feet. However, some postoperative complications may occur 3–6 months after subtalar arthroereisis, and few studies have explored plantar pressure and lower limb muscle activation patterns during this period.

**Methods:**

Twenty adolescents with flexible flatfoot deformities who underwent subtalar arthroereisis were enrolled in this study. The plantar areas of all patients were divided into eight regions, and the average standing pressure and peak pressure during the gait cycle were compared before and three months after surgery. Surface electromyography (sEMG) of the tibialis anterior (TA), peroneus longus (PL), and medial gastrocnemius (MG) muscles was simultaneously measured during a single gait cycle.

**Results:**

All patients were able to walk in their shoes at 3 months postoperatively. After surgery, while standing, the average pressure on the lesser toes, lateral forefoot, and lateral midfoot increased significantly (*p* < 0.05). In contrast, the pressure of the hallux region, medial forefoot, medial midfoot, medial hindfoot, and lateral hindfoot decreased significantly (*p* < 0.05). During the gait cycle, the peak pressure in the lesser toes, lateral forefoot, lateral midfoot, and lateral hindfoot increased significantly (*p* < 0.05), whereas that in the hallux, medial forefoot, medial midfoot, and medial hindfoot decreased significantly (*p* < 0.05). The maximum lateral displacement of the center of pressure (COP) decreased from 3.81 ± 0.56 cm preoperatively to 3.59 ± 0.41 cm postoperatively. The maximum longitudinal displacement decreased from 21.07 ± 3.96 cm to 19.37 ± 3.08 cm (*p* < 0.05), and the COP trajectory curve shifted laterally. During the gait cycle, the peak activation percentage of TA significantly decreased postoperatively, that of the PL significantly increased after surgery, and that of the MG significantly decreased. The integral percentage of TA activation was significantly reduced postoperatively. The integral percentage of PL activation was significantly higher than that at the preoperative stage. Additionally, the integral percentage of MG activation was significantly lower than that of the preoperative value. (all *p* < 0.05).

**Conclusion:**

This study found that plantar pressure shifted laterally during the early postoperative period. Such changes in plantar pressure distribution may compensate for alterations in lower limb muscle activation patterns, which may potentially contribute to postoperative plantar pain or painful peroneal muscle spasm. Therefore, monitoring plantar pressure distribution and muscle activation in the early postoperative period is recommended.

## Introduction

**Figure 1:**
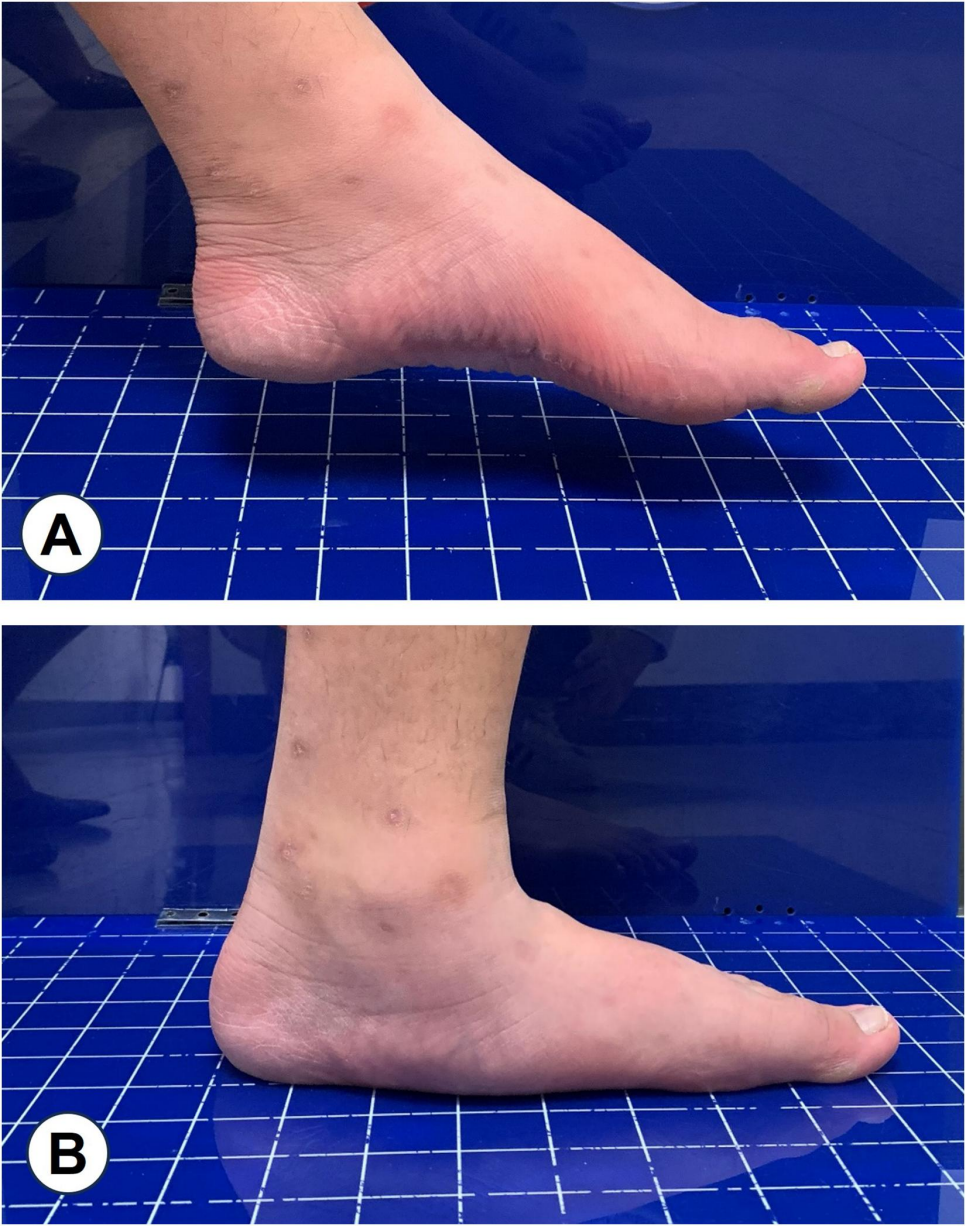
Flexible flatfoot: **A**, normal arch height under non-weight-bearing conditions; **B**, collapse of the arch under weight-bearing conditions.

Flatfoot is characterized by calcaneal valgus, adducted talus with plantar flexion, and medial longitudinal arch collapse, which can lead to symptoms such as muscle fatigue, foot pain, and abnormal gait (1). This condition can be categorized into two types: flexible and rigid, depending on whether the arch maintains its normal height in a non-weight-bearing state (2). Flexible flatfoot is characterized by a reduction in arch height when weight is applied, with the arch returning to its normal position when the weight is removed (Figure 1), whereas rigid flatfoot involves arch collapse regardless of the weight-bearing status. In adolescents, approximately 41.6% of children attending a pediatric orthopedic clinic in Saudi Arabia were diagnosed with flexible flatfoot (318 of 2,321 cases, aged ≤12 years) (3). A large kindergarten-based survey in Austria involving 835 preschool-aged children (3–6 years) reported a prevalence of 44%, with rates decreasing from 54% at age 3%–24% at age 6 (4). Similarly, in China Taiwan, epidemiological data indicate that the prevalence decreases from around 54%–57% at ages 2–3 to about 21%–24% at ages 5–6, and further to approximately 15% by age 10 (5, 6). In Shaanxi Province, China, a cross-sectional study of 1,059 school-aged children (6–13 years) in Xi’an found that flexible flatfoot prevalence dropped from 39.5% at age 6 to 11.8% at age 12, stabilizing thereafter (7).

Flexible flatfoot therapy aims to alleviate symptoms and prevent future disabilities. Sullivan et al. did not recommend early intervention for flexible flatfoot (8). Previous studies have explored the impact of custom orthotic insoles on children with flexible flatfoot (9–13). However, surgery is recommended if the patient continues to experience arch collapse, pain around the ankle, and foot discomfort after at least six months of conservative treatment (14). Although osteotomy can effectively correct foot malalignment, it is relatively complex and is associated with a variety of potential postoperative complications (15). In recent years, subtalar arthroereisis has been shown to reduce excessive pronation of the foot and correct foot arch deformities by implanting a prosthesis into the subtalar joint. This approach is gradually gaining popularity in the treatment of flexible flatfoot owing to its minimal trauma, short postoperative recovery time, and compatibility with future surgical interventions (16–18).

Current studies primarily focus on the medium-term assessment of subtalar arthroereisis efficacy. Some scholars have reported significant improvement in imaging and function after surgery, and some patients have also observed arch elevation and hindfoot alignment correction after surgery (17, 19, 20). Richter and Zech utilized pedography to evaluate patients before the procedure and at implant removal, revealing sustained corrective benefits after implant removal and up to six months later (21). Xu et al. evaluated severe adolescent flexible flatfoot using calcaneal Z osteotomy combined with STA in a small cohort of only 16 patients, without assessment of plantar pressure or neuromuscular function (22). Vogt et al. included pedobarographic analysis in 73 children (113 feet), but electromyographic (EMG) data were not collected, and follow-up assessments were conducted mainly at implant removal or two years postoperatively rather than during the early postoperative period (23). Similarly, Wang et al. reported mid-term outcomes of Talar-Fit implants in a cohort, yet their follow-up emphasized radiographic correction and complications rather than short-term functional adaptations (18).

Plantar pressure analysis provides an objective biomechanical assessment of foot load redistribution and arch function, directly reflecting how surgical correction influences weight-bearing and gait mechanics. Evaluating changes in plantar pressure can therefore offer valuable insight into early functional recovery and compensatory adaptations following STA. However, plantar pressure alone cannot fully capture the neuromuscular mechanisms underlying postoperative improvement. The restoration of the medial longitudinal arch depends not only on passive structural correction but also on dynamic muscle control, particularly involving the tibialis posterior, peroneus longus, and intrinsic foot muscles. Previous studies have demonstrated altered EMG patterns in patients with flexible flatfoot, such as delayed activation of the tibialis posterior and compensatory overactivity of the peroneal muscles. Which may gradually normalize following corrective surgery or orthotic intervention (24, 25). Furthermore, pedobarographic studies on children undergoing subtalar arthroereisis have shown early postoperative changes in plantar load distribution, supporting the concept that neuromuscular adaptation contributes to improved hindfoot stability after surgery (23).

Therefore, this study aimed to investigate changes in plantar pressure distribution as the primary outcome and muscle activation patterns as the secondary outcome during the early postoperative phase after subtalar arthroereisis in adolescents with flexible flatfoot. This combined pedobarographic and electromyographic approach was designed to comprehensively characterize both biomechanical and neuromuscular adaptations, providing a more integrated understanding of early functional recovery after STA.

## Methods

### Participants

This study included 20 children with flexible flat feet who underwent subtalar arthroereisis. Participants were prospectively and consecutively recruited during hospitalization for surgery. Potential candidates were identified from the inpatient surgical list, and recruitment was conducted by the research team through direct communication with patients and their guardians before the operation. All participants and their guardians received detailed information about the study objectives, procedures, and potential risks, and provided written informed consent prior to enrollment. This study adhered to the principles of the Declaration of Helsinki and was approved by the institutional ethics committee (Approval No. 2014-056).

Eligible patients were 10–18 years of age and were diagnosed with flexible flatfoot based on radiographic examination and the navicular drop test. The diagnostic criteria followed established standards in the literature: a Meary angle (talus–first metatarsal angle) <–4° and a calcaneal pitch angle <20° on weight-bearing lateral radiographs, both indicative of arch collapse (26). And a navicular drop greater than 10 mm between non–weight-bearing and weight-bearing positions, which has been widely accepted as abnormal and associated with excessive pronation and flexible flatfoot (27, 28).

All patients had foot symptoms, including pain and soreness, which were not significantly relieved after conservative treatment for at least six months. All participants underwent unilateral subtalar arthroereisis as described by Wang, using the Talar-Fit implant (OsteoMed, USA) (18). Detailed anthropometric information of the participants is shown in Table 1. All participants underwent weight-bearing anteroposterior and lateral foot radiographic examinations before surgery. Physical examination revealed that the foot arch disappeared during weight-bearing and reappeared during non–weight-bearing.

**Table 1:** The anthropometric data of the participants.

Characteristic	Value
Age (year)	13.00 ± 2.08 (10–18)
Gender (Male: Female)	14:6
Height (cm)	160.95 ± 6.24 (150–170)
Weight (kg)	53.55 ± 7.76 (40–65)
Affected foot (Left: right)	8:12

A formal sample size calculation was not performed because this study was designed as an exploratory investigation to characterize postoperative changes in plantar pressure distribution and muscle activation patterns after subtalar arthroereisis in children with flexible flatfoot. The number of participants (*n* = 20) was determined by the availability of eligible surgical cases during the study period and is comparable to previous biomechanical and pedobarographic studies evaluating subtalar arthroereisis, which typically enrolled 10–40 patients (15, 29, 30). These studies demonstrated that similar sample sizes were sufficient to detect clinically relevant changes in foot loading patterns and muscle activity following surgery. Therefore, the present sample size was considered adequate for exploratory analyses and hypothesis generation.

### Experimental procedure

**Figure 2:**
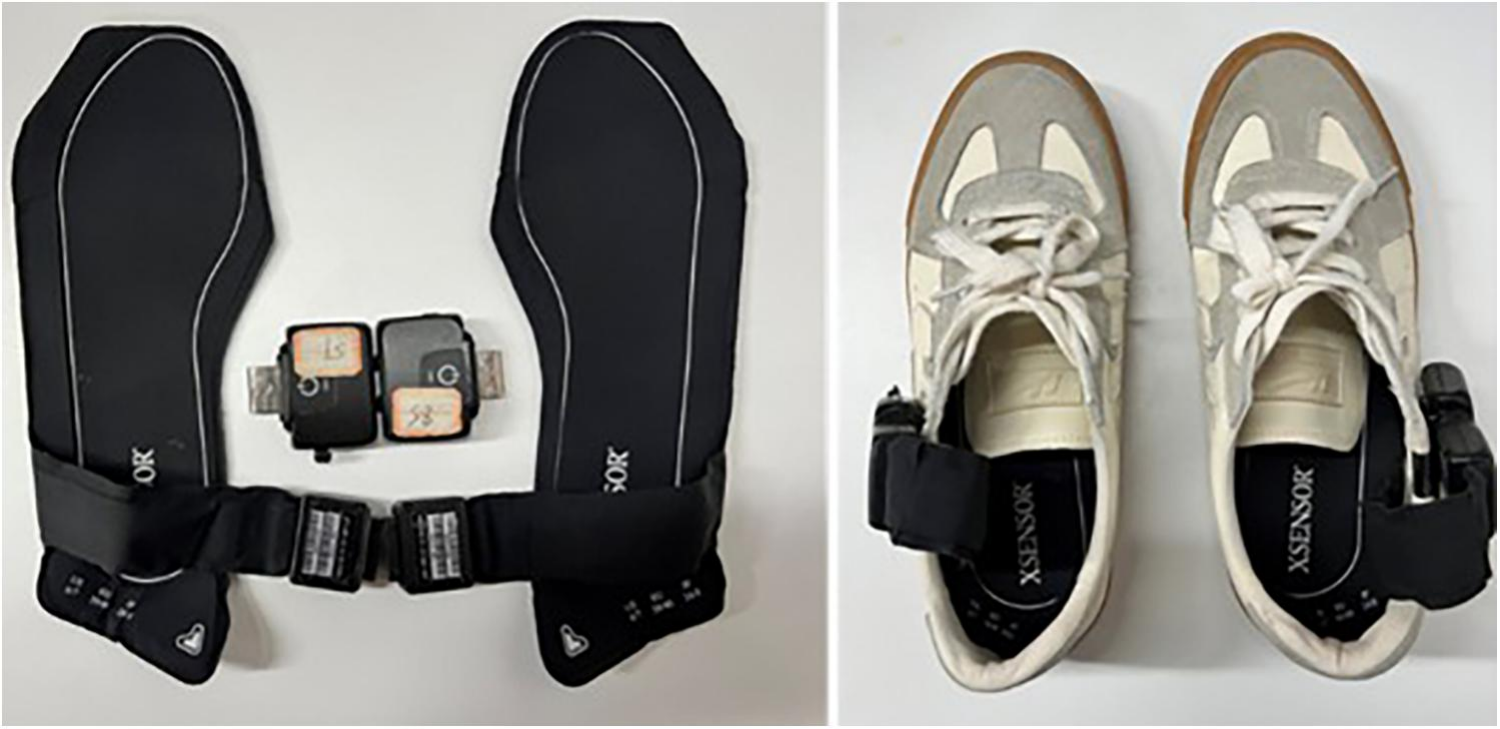
Install and connect XSENSOR X4 insole foot pressure and gait measurement system.

Plantar pressure was quantified using the XSENSOR X4 insole Foot Pressure and Gait Measurement System (XSENSOR Technologies, Canada). This system features a sampling rate of 150 fps and incorporates 230 sensors per insole. To minimize variability related to footwear, all participants wore standardized test shoes provided by the research team (Figure 2). The shoes were available in multiple sizes to ensure proper fit, and were manufactured with a flat rubber outsole and a canvas upper, ensuring uniform sole hardness and upper material across all participants. An in-shoe pressure sensor of the corresponding size was then inserted, and the device was calibrated by zeroing the data acquisition unit. Prior to the walking task, the participants were instructed to maintain a static standing position for a minimum of 15 s, during which plantar pressure readings were recorded.

Surface electromyography (sEMG) signals from the lower limbs were recorded using a Portable Lab device (Noraxon, United States). Before EMG data collection, the EMG sensor attachment site was prepared using standard skin preparation procedures, which involved shaving the leg hair, eliminating surplus cuticle, and cleansing the skin with an alcohol-based cotton ball. The calf muscle groups confirmed to be associated with flatfoot in previous studies were selected for testing (24, 31). The electrode sites of tibialis anterior (TA), peroneus longus (PL) and medial gastrocnemius (MG) were placed according to the guidelines (32). The posterior tibialis muscle, although crucial for medial arch support, was not assessed because its deep anatomical location requires needle EMG, which is invasive and not feasible in this pediatric population. Therefore, we focused on superficial muscles that can be reliably evaluated using surface electrodes.

After the pretest readiness procedure, electromyography (EMG) measurements and maximal voluntary isometric contraction (MVIC) were used to normalize the EMG amplitude parameters. At the completion of each test session, the MVIC was performed three times per muscle, consisting of a gradual and continuous 2 s accumulation followed by a maximum 2 s effort. Each participant was instructed to perform maximal contractions against the tester's resistance and was given verbal encouragement to do so. Resistance exercises included pronation-peroneus longus, dorsiflexion-tibialis anterior, and plantar flexion (knee extension) -medial gastrocnemius exercises. The participants sat on a bench while the MVIC was performed on the tibialis anterior and peroneal muscles. For the medial gastrocnemius mvic, participants were seated on the floor with their back against a wall to ensure that participants did not slide backwards during contractions (24, 33). Following each MVIC recording, participants were advised to take a 30-s rest period.

Before the official gait assessment, the participants were given two opportunities to warm up. The warm-up phase allowed the patients to walk without constraints on their stride length, speed, or frequency. After the warm-up, the participants were instructed to walk at a self-selected pace and frequency. Each subject was required to complete two walks of approximately 10 m in distance. EMG and plantar pressure data were continuously collected during walking trials. To minimize the influence of acceleration and deceleration, the first and last two steps of each trial were excluded. Only the steady-state gait cycles from the middle portion of each walk were used for analysis.

**Figure 3:**
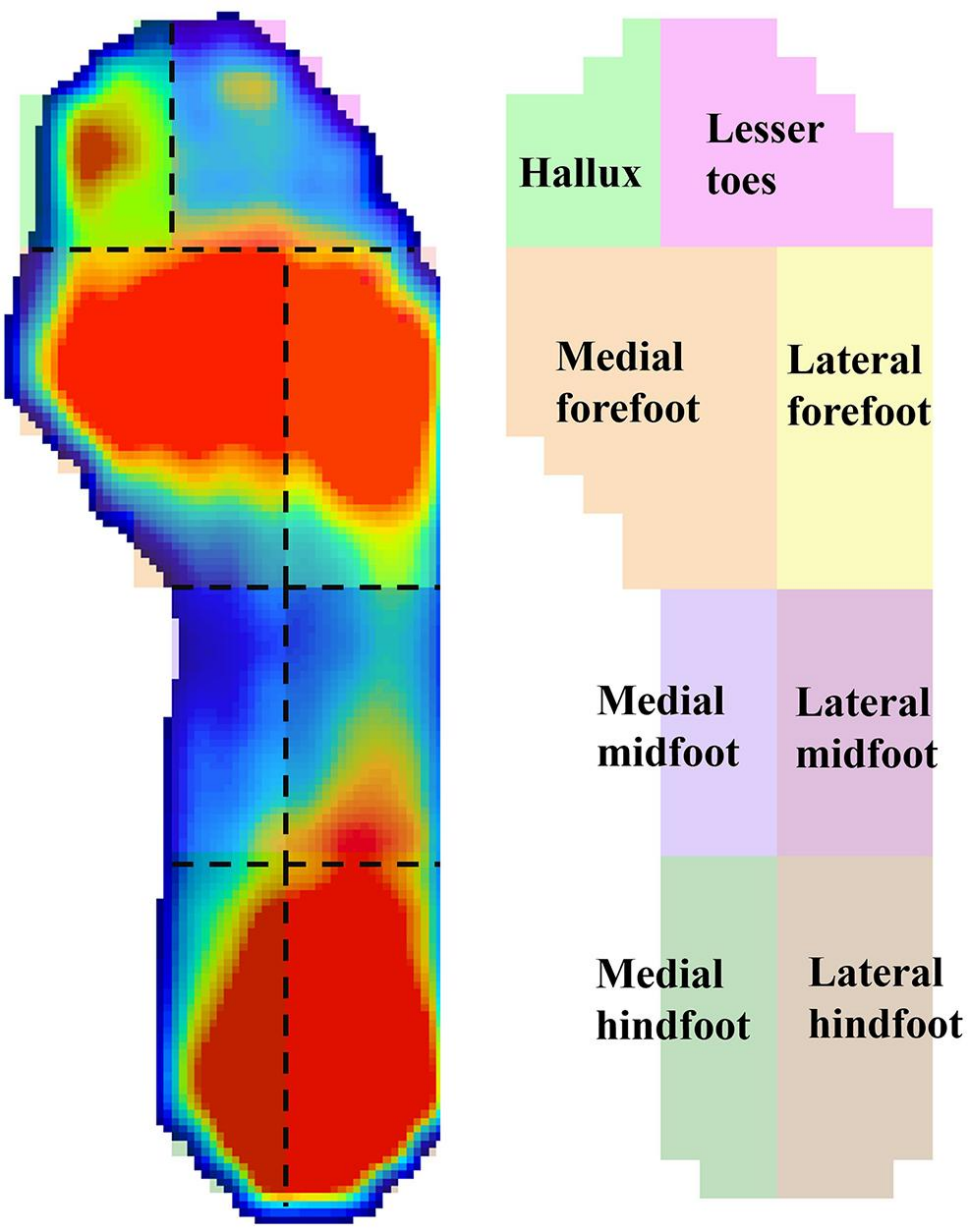
The plantar pressure was divided into eight regions during the analysis.

The plantar pressure data of the participants were inputted into the XSENSO Pro Foot & Gait analysis software. The XSENSOR Pro software automatically identified gait cycles from the in-shoe pressure time series. Plantar region masks were adapted from Schmidt et al. with a modification where the forefoot was consolidated from three masks to two (medial/lateral) by merging the central forefoot along the foot midline, in order to emphasize mediolateral load shifts and limit multiple comparisons (34). Accordingly, eight regions were analyzed: medial hindfoot, lateral hindfoot, medial midfoot, lateral midfoot, medial forefoot, lateral forefoot, hallux, and lesser toes (Figure 3). The mean pressure during standing, peak pressure during the gait cycle, and maximum transverse and longitudinal displacements of the center of pressure (COP) were analyzed, and the COP trajectories were plotted.

The unprocessed EMG data were input into Matlab R2022a software and then subjected to bandpass filtering (20–450 Hz), full-wave rectification, and linear envelope extraction. The EMG signals recorded during the MVIC were used to standardize the EMG activity across the entire gait cycle. Following normalization, the EMG data for each gait cycle were adjusted to 1,000 values. Subsequently, the muscle activation percentages were computed for each individual.

### Statistical analysis

SPSS 20.0 (SPSS, USA) was used for statistical analysis. Measurement variables are expressed as mean ± standard deviation. The Kolmogorov–Smirnov test was applied to assess normality. For normally distributed data, paired sample *t*-tests were used to compare preoperative and postoperative values of plantar pressure, COP displacement, and lower limb muscle activation parameters. To reduce the risk of type I error due to multiple comparisons, *p*-values were adjusted using the Holm–Bonferroni method within each outcome category. A two-sided adjusted *p* < 0.05 was considered statistically significant.

## Results

All patients were instructed to remove their cast six weeks post-surgery and gradually began to walk with weight-bearing. Postoperative assessment was conducted three months after the surgical procedure, allowing for a one-week error margin. In the initial postoperative phase, three patients reported tarsal sinus pain, one had slow wound healing, and two had slight discomfort during weight bearing. Symptoms were relieved after symptomatic treatment, and all patients were able to walk in shoes 3 months postoperatively.

**Figure 4:**
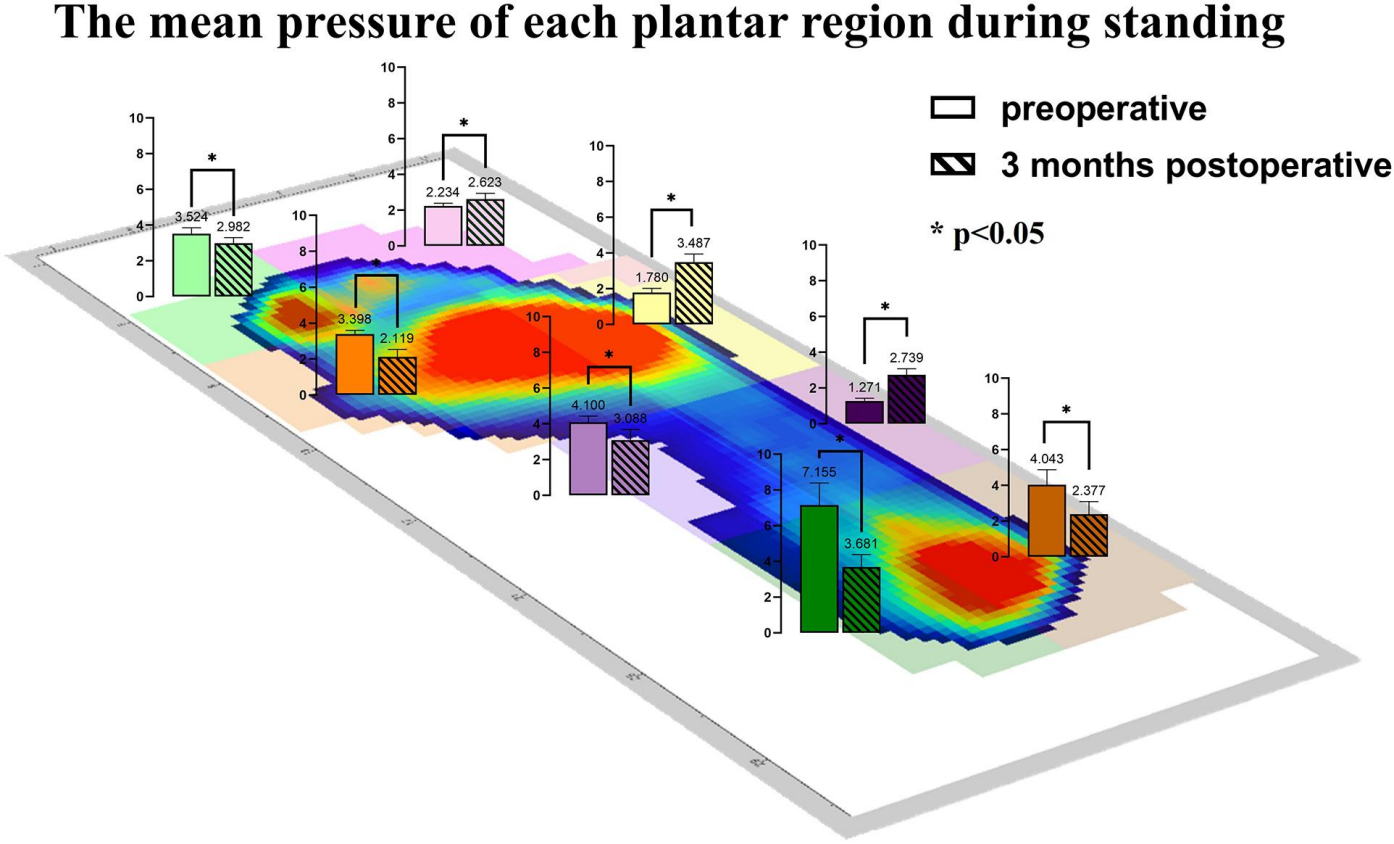
The average pressure of each plantar region during standing before and after the operation.

**Figure 5:**
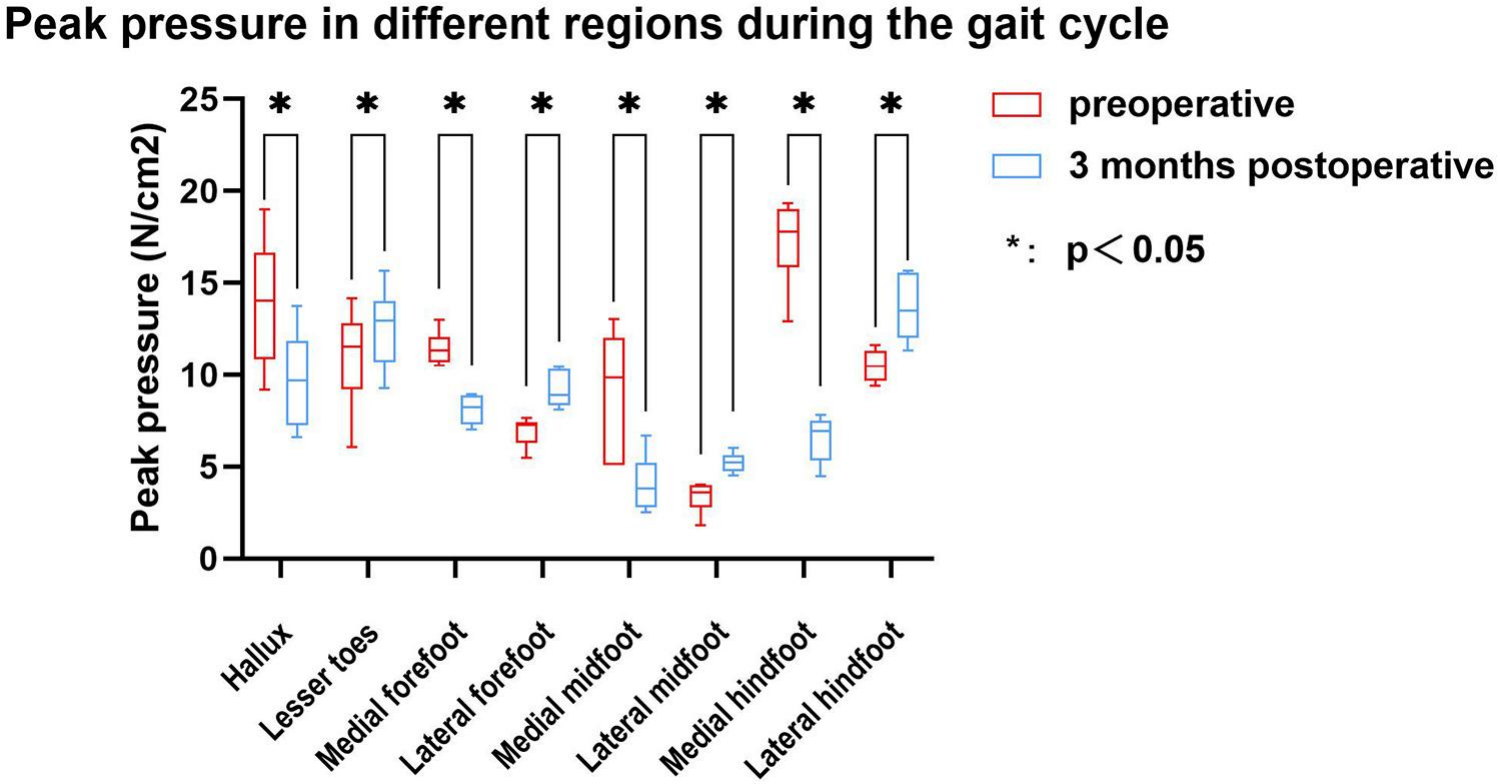
The peak pressure in the gait cycle in different regions before and after surgery.

**Figure 6:**
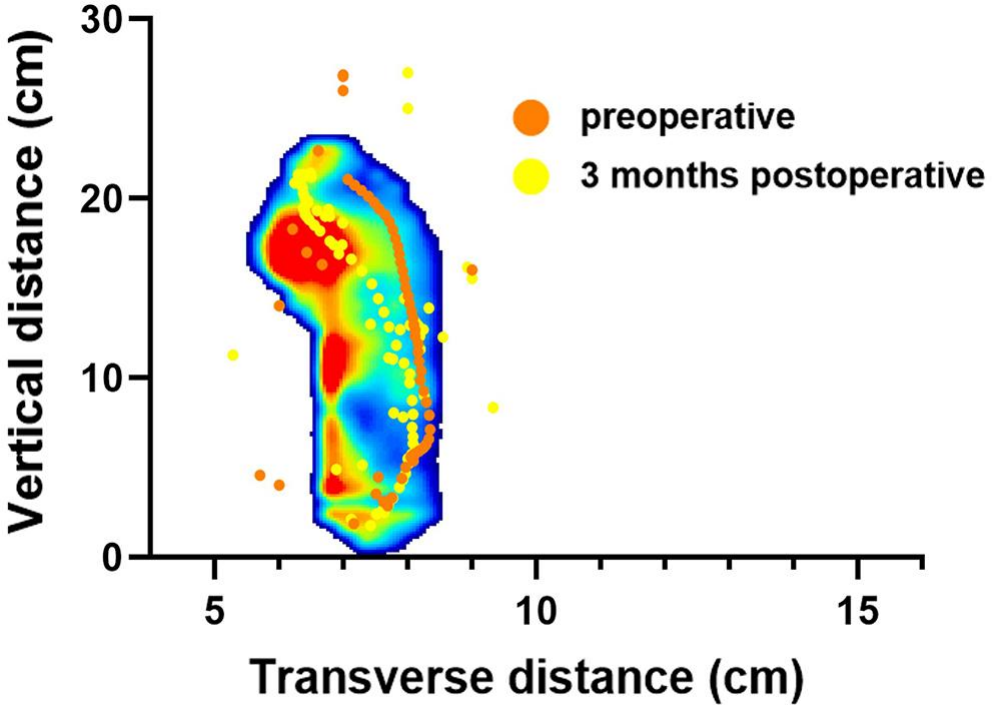
One patient's center of pressure trajectory during gait cycle before and after surgery.

The study compared the mean pressure on different areas of the foot while standing (Figure 4), and the results are shown in Table 2. After surgery, the pressure on the lesser toes, lateral forefoot, and lateral midfoot increased significantly (*p* < 0.05). In contrast, the hallux, medial forefoot, medial midfoot, medial hindfoot, and lateral hindfoot pressures were significantly decreased (*p* < 0.05).During the gait cycle, the results presented in Table 3 show that the peak pressure in the lesser toes, lateral forefoot, lateral midfoot, and lateral hindfoot increased significantly (*p* < 0.05), whereas in the hallux region, medial forefoot, medial midfoot, and medial hindfoot decreased significantly (*p* < 0.05; Figure 5). In the interim, there was a reduction in the maximum transverse displacement of the COP from 3.81 ± 0.56 cm to 3.59 ± 0.41 cm. Similarly, the maximum longitudinal displacement decreased from 21.07 ± 3.96 cm to 19.37 ± 3.08 cm, with a statistically significant difference observed (*p* < 0.05, Table 4). The COP trajectory image indicated a decrease in the patient's COP displacement range and a lateral shift in the COP trajectory curve (Figure 6).

**Table 2:** Average plantar pressure in different plantar regions during static standing.

Regions	Paired (mean ± SD) (unit: N/cm^2^)		Value of difference	*P* value (unadjusted)	*P* value (Holm–Bonferroni adjusted)
	Preoperative	Postoperative			
Hallux	3.52 ± 0.33	2.98 ± 0.33	0.54 ± 0.58	0.016*	0.033*
Lesser toes	2.23 ± 0.16	2.62 ± 0.32	−0.39 ± 0.32	0.004*	0.012*
Medial forefoot	3.40 ± 0.20	2.11 ± 0.42	1.28 ± 0.36	0.000*	0.000*
Lateral forefoot	1.78 ± 0.24	3.49 ± 0.46	−1.71 ± 0.35	0.000*	0.000*
Medial midfoot	4.10 ± 0.34	3.09 ± 0.60	1.01 ± 0.61	0.001*	0.003*
Lateral midfoot	1.27 ± 0.16	2.74 ± 0.34	−1.47 ± 0.61	0.000*	0.000*
Medial hindfoot	7.15 ± 1.23	3.68 ± 0.70	3.48 ± 1.24	0.000*	0.000*
Lateral hindfoot	4.04 ± 0.84	2.38 ± 0.72	1.67 ± 1.07	0.001*	0.003*

* *p* < 0.05 (statistically significant).

**Table 3:** Peak pressure in different plantar regions during the gait cycle.

Regions	Paired (mean ± SD) (unit: N/cm^2^)		Value of difference	*P* value (unadjusted)	*P* value (Holm–Bonferroni adjusted)
	Preoperative	Postoperative			
Hallux	13.92 ± 3.42	9.73 ± 2.63	4.19 ± 0.92	0.000*	0.001*
Lesser toes	10.99 ± 2.76	12.58 ± 2.17	−1.60 ± 0.86	0.006*	0.025*
Medial forefoot	11.44 ± 0.88	8.11 ± 0.79	3.34 ± 1.38	0.002*	0.012*
Lateral forefoot	6.92 ± 0.80	9.19 ± 0.99	−2.27 ± 1.53	0.015*	0.025*
Medial midfoot	9.09 ± 3.34	4.08 ± 1.60	5.02 ± 2.20	0.003*	0.013*
Lateral midfoot	3.35 ± 0.86	5.22 ± 0.55	−1.87 ± 1.05	0.007*	0.025*
Medial hindfoot	17.26 ± 2.31	6.53 ± 1.27	10.73 ± 3.10	0.000*	0.003*
Lateral hindfoot	10.49 ± 0.91	13.63 ± 1.75	−3.14 ± 1.86	0.009*	0.025*

* *p* < 0.05 (statistically significant).

**Table 4:** The maximum transverse and longitudinal displacement of the COP during the gait cycle.

Variable	Preoperative (mean ± SD, cm)	Postoperative (mean ± SD, cm)	*P* value (unadjusted)	*P* value (Holm–Bonferroni adjusted)
COPdx	3.81 ± 0.56	3.59 ± 0.41	0.014*	0.014*
COPdy	21.07 ± 3.96	19.37 ± 3.08	0.004*	0.009*

COPdx: the maximum transverse displacement of the COP, COPdy: The maximum longitudinal displacement of the COP.

* *p* < 0.05 (statistically significant).

**Figure 7:**
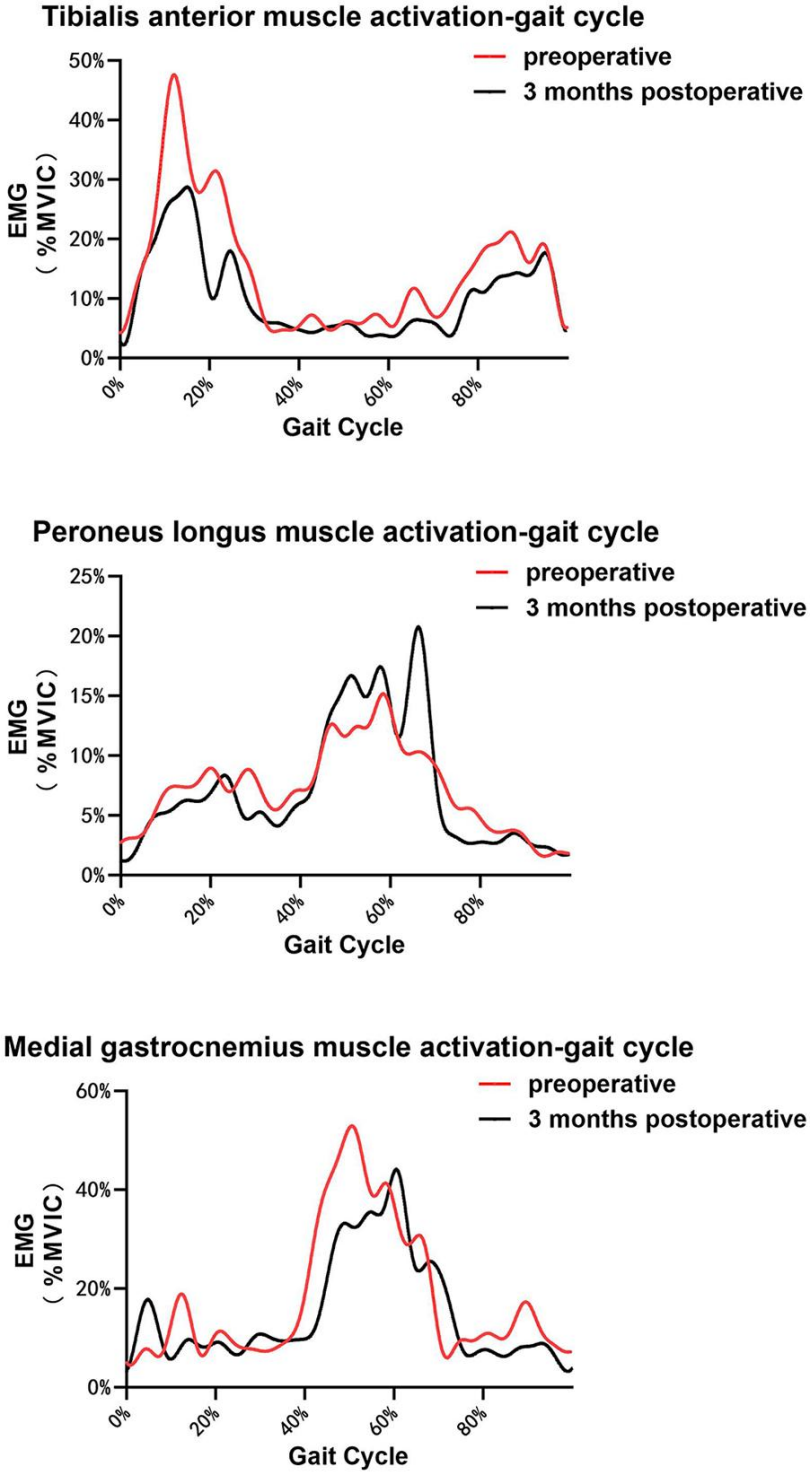
EMG values of TA, PL, and MG (medial gastrocnemius) in a single gait cycle for all the participants. (Please note that the curves are slightly different from the actual results because they are derived from a single gait cycle for each participant. The main findings are intended to be indirectly illustrated with this figure.).

This study also compared the muscle activation levels (Table 5), comprising six pairs of data. Following the operation, the average peak activation percentage of the TA was 39.90% ± 5.41%, significantly lower than the preoperative level (*p* < 0.05). Conversely, the average peak activation percentage of the PL was 30.21 ± 2.06%, which was significantly higher postoperatively (*p* < 0.05). The mean peak activation percentage of the MG after surgery was 58.25% ± 3.72%, which was significantly lower than that before surgery (*p* < 0.05). The average integration percentage of TA activation was significantly lower than that of the preoperative value. Similarly, the average integration percentage of PL activation was significantly higher than that at the preoperative stage. Additionally, the average integration percentage of MG activation was significantly lower than the preoperative value (*p* < 0.05). The mean activation level observed in the lower limb muscles during the gait cycle is shown in Figure 7.

**Table 5:** Peak and integral values of calf muscle activation during the gait cycle.

Parameters	Paired (mean ± SD) (unit: MVIC%)		Value of difference	*P* value (unadjusted)	*P* value (Holm–Bonferroni adjusted)
	Preoperative	Postoperative			
TA activation peak percentage	52.22 ± 6.09	39.90 ± 5.41	−12.31 ± 6.13	0.000^*^	0.000*
PL activation peak percentage	26.99 ± 1.50	30.21 ± 2.06	3.22 ± 1.32	0.000^*^	0.000*
MG activation peak percentage	83.26 ± 4.48	58.25 ± 3.72	−25.01 ± 4.00	0.000^*^	0.000*
TA activation percentage integration	18.39 ± 1.98	16.51 ± 1.79	−1.88 ± 0.79	0.000^*^	0.000*
PL activation percentage integration	7.98 ± 0.70	8.80 ± 0.28	0.82 ± 0.58	0.001^*^	0.001*
MG activation percentage integration	18.11 ± 3.37	14.28 ± 2.24	−3.83 ± 1.90	0.000^*^	0.000*

* *p* < 0.05 (statistically significant).

## Discussion

There has been an increasing trend in the prevalence of flatfoot among adolescents over the years. The presence of flexible flat feet can affect the postural development and motor skills of children. And it may lead to muscle fatigue and discomfort in the lower extremities, whicn may interfere with daily activities (35–37). Conservative treatment is usually the first choice for the treatment of flexible flatfoot (11, 38, 39). When there is no significant relief of symptoms after at leastsix6 months of conservative treatment, further surgical treatment is recommended. Subtalar arthroereisis is becoming increasingly popular as a minimally invasive treatment option. Studies have shown that the prognosis of this type of surgery in adolescent patients is usually better than that in adult patients, which some scholars believe may be due to the higher arch extensibility and adaptability of adolescent patients (5, 20, 40–42).

Pedobarography is a reliable method for the objective and qualitative evaluation of pressure distribution changes in various plantar regions. The variation in plantar peak pressure distribution reflects the structural and functional changes in the foot. Local high pressure is associated with hypoesthesia and skin damage (34, 43). Previous studies have shown that flatfoot is often accompanied by medial plantar pain and wear (44, 45). This agrees with our preoperative measurements of plantar pressure distribution in our patients, and our results showed that there was a greater peak pressure in the medial plantar area before surgery during static and dynamic activities.

During the postoperative gait cycle, we observed significant increases in the peak pressures of the lateral plantar regions in the early postoperative period. For example, the peak pressure of the lateral forefoot increased from 6.92 ± 0.80 N/cm^2^ preoperatively to 9.19 ± 0.99 N/cm^2^ postoperatively (*p* < 0.05), the lateral midfoot increased from 3.35 ± 0.86 to 5.22 ± 0.55 N/cm^2^ *p* < 0.05), and the lateral hindfoot increased from 10.49 ± 0.91 to 13.63 ± 1.75 N/cm^2^ (*p* < 0.05). These findings indicate a redistribution of plantar loading toward the lateral side after subtalar arthroereisis. Therefore, strengthening the protection of the lateral plantar region in the early postoperative period is recommended to minimize potential complications caused by abnormal pressure. Our findings are consistent with Franz et al, who also observed a postoperative shift of plantar load from the medial to the lateral regions of the mid- and forefoot (29). However, Franz et al. primarily reported changes in force–time integrals and contact areas, showing that the lateral midfoot impulse was even higher than in healthy controls, suggesting a potential overcorrection. In contrast, our study focused on peak plantar pressures and dynamic COP trajectories, demonstrating significant postoperative increases in lateral forefoot, midfoot, and hindfoot pressures as well as a lateral COP shift without hallux engagement at toe-off.

Interestingly, in static standing, the pressure in the lateral hindfoot region decreased postoperatively, whereas it increased during the gait cycle. This discrepancy likely reflects the distinct biomechanical demands of static and dynamic conditions. During quiet standing, the subtalar implant restricts excessive eversion and reorients the calcaneus toward a more neutral alignment, leading to a more centralized heel loading and reduced relative pressure on the lateral hindfoot. In contrast, during walking, the restored hindfoot alignment enables a more physiological lateral-to-medial weight transfer through the foot tripod mechanism. The lateral hindfoot thus becomes more actively engaged during heel strike and load absorption, resulting in higher peak pressure during gait. These complementary effects suggest that subtalar arthroereisis not only corrects abnormal pronation during static standing but also restores a more balanced and physiological load transition during dynamic activities, thereby improving both postural stability and gait function.

Our results showed a significant decrease in tibialis anterior (TA) activation, a significant increase in peroneus longus (PL) activation, and a significant decrease in medial gastrocnemius (MG) activation. Notably, the observed increase in PL activation was consistent with the findings of Saeedi et al. (31), who reported a 7% increase in PL activity in a patient with flexible flatfoot using a modified foot orthosis with a heel cup design. However, in their study the changes in TA (–1%) and MG (+1%) activity were minimal and not significant, whereas in our cohort both muscles showed marked postoperative reductions. This discrepancy may be explained by the different interventions: Saeedi evaluated an external orthosis that primarily provided passive arch support and therefore mainly influenced PL activity without substantially altering overall gait mechanics. In contrast, our approach involved direct subtalar arthroereisis with screw implantation, which induced more profound morphological correction and plantar pressure redistribution. These biomechanical alterations may have reduced the demand on TA during swing initiation and decreased MG involvement during push-off, thereby leading to the significant reductions observed in our study. Similarly, Reeves et al. concluded that foot orthoses and footwear modifications that elevate or support the medial arch tend to reduce tibialis posterior activity and concomitantly increase PL activation (46). our study involved surgical realignment of the subtalar joint, which produced structural changes in foot morphology and a lateral shift in plantar loading. This morphological alteration likely induced a compensatory increase in PL activation, in agreement with the mechanism proposed by Reeves.

In previous studies, unusual forefoot supination has been documented in patients undergoing subtalar arthroereisis and typically diminishes within approximately six months; at one year, muscle activation patterns may resemble those of healthy controls (15, 47). Against this longitudinal backdrop, Although our study only examined outcomes at 3 months, the similarity in trends suggests that gradual changes in foot morphology may be accompanied by synchronous adaptations in muscle activation. Nevertheless, this interpretation should be considered with caution, and longer-term follow-up studies are required to verify whether the early changes we observed persist and ultimately converge toward the normalization reported by Caravaggi et al. (15).

Mechanistically, we posit that postoperative changes in foot morphology alter plantar pressure distribution and then modulate muscle activation through proprioceptive and load-sensitive reflex pathways. In particular, increased loading in the lateral plantar regions may enhance afferent input from plantar fascia and the subtalar joint, eliciting a compensatory rise in peroneus longus activation. This interpretation is supported by reports that the sinus tarsi—rich in mechanoreceptors—acts as a proprioceptive center of the subtalar joint; pathological afferent input from this region can trigger abnormal peroneal spasm and can be alleviated when sensory input is normalized (48). While such compensation may help counteract abnormal load distribution, it may also predispose to painful peroneal muscle spasm after surgery, as described by Vogt et al. (23) Converging evidence from intervention studies further supports a pressure-to-activation pathway: anti-pronation taping and customized foot orthoses can modify plantar loading and influence lower-limb muscle activity by altering afferent input from plantar and articular mechanoreceptors (49, 50). For instance, inverted-angle foot orthoses acutely redistribute loads in flexible flatfoot—reducing medial forefoot/rearfoot peaks while increasing medial midfoot loading and contact area during gait (51); augmented Low-Dye taping and ankle bracing reduce walking EMG amplitudes of tibialis posterior, peroneus longus, and tibialis anterior (52); and Kinesio taping of tibialis posterior or peroneus longus yields immediate improvements in foot posture, with peroneus-longus taping improving dynamic balance and widening the gait line (53). Based on these findings, the potential use of foot orthoses or elastic bandages could be considered in early rehabilitation, although future studies are required to confirm their effectiveness.

### Limitations

This study had several limitations. We chose a 3-month postoperative follow-up to assess alterations in pressure distribution during the early stage of recovery; however, individual variations in patient recovery may have introduced abnormal gait patterns due to incomplete restoration of walking function. Surface electromyography was performed with skin electrodes, which limited our ability to evaluate deeper muscles such as the posterior tibialis. Because the posterior tibialis lies beneath several layers of soft tissue, its signals are attenuated with surface electrodes. Although needle electromyography has been used in previous studies (46), we deliberately avoided this technique because of its invasive nature and associated ethical concerns, in addition to the higher cost and potential risk of subject discomfort or injury. Therefore, posterior tibialis activation could not be directly assessed in this study. Additionally, different muscles may have required different thresholds for signal acquisition, although all EMG data were normalized to minimize potential bias. Another important limitation is the relatively small sample size (*n* = 20) and the single-center design, which may restrict the statistical power of the analyses and limit the generalizability of our findings. Future multicenter studies with larger cohorts and longer follow-up are warranted to validate and extend these results.

## Conclusion

Through early postoperative follow-up of patients treated with subtalar arthroereisis, this study found that plantar pressure shifted laterally in the early postoperative period. This change in plantar pressure distribution may potentially contribute to alterations in the activation patterns of some lower limb muscles. Such abnormal pressure distribution and increased muscle activation may be related to the development of postoperative plantar pain or painful peroneal muscle spasms. Therefore, monitoring plantar pressure distribution and muscle activation in patients during the early postoperative period is recommended. The use of foot orthoses or elastic exercise bandages could be considered during early rehabilitation to help reduce abnormal plantar pressure and peroneal muscle activation, thereby potentially assisting patients in completing early postoperative recovery.

## Data Availability

The original contributions presented in the study are included in the article/Supplementary Material, further inquiries can be directed to the corresponding author.
